# Disease in the Antrum

**Published:** 1893-08

**Authors:** J. B. Van Fossen


ARTICLE IV.

### DISEASE IN THE ANTRUM.

J. B. VAN FOSSEN, D. D. S.

Read before the Detroit Dental Society, March 13th, 1893.

This curious space is located in the body of the upper maxilla. There are several theories in regard to its real office. Some claim it is to make the bones of the face a little lighter; others, that it is a kind of sounding apparatus to give pitch to the voice; and still others claim it is for the greater distribution of the olfactory nerve to aid the sense of smell. One thing is certain; its disease is very disagreeable for dentist and patient.

This curious space has but one opening. This leads to the nose. Sometimes the roots of the teeth puncture the floor of the antrum, and in ulceration are apt to make a disturbance.

The case to which I wish to call your attention, was one I had in practice a few months ago. The patient was a lady, about forty-five years of age. The first time I saw her was during the summer, and at that time she complained of a dull, heavy pain on the right side of her face, just below the eye. There seemed to be a slight swelling. I examined her mouth and advised her to have all the teeth in the upper jaw removed, which was done. The tooth that seemed to have made the trouble, was the first upper molar, and I remember I had some difficulty in taking it out. The tooth was free from decay, and the only one that was in a good state of preservation. In six weeks she called again. Her face was badly swollen, and she informed me she had been confined to her bed most of her time since I removed her teeth; her physician treating her for I don't know how many things. On examining her mouth, I found the opening where I had removed the molar tooth had not healed, and there was a small teat-like substance hanging from the opening, resembling gristle in structure. I removed it with some difficulty, but there did not seem to be any discharge. I, at once, mistrusted the cause, but, as I had nothing to treat it with, I plugged up the opening with a piece of cotton rolled hard and moistened with oil of eucalyptus, and dismissed the patient, requesting her to call the next day. On her return I took one of the largest sized "rose burs" and drilled through till I struck the floor of the antrum—making a good free opening. I then had the patient bend well forward over a large washbowl, and with a small rubber syringe I washed the cavity thoroughly, using as much force as I could in throwing the water into the antrum. I soon had a good discharge, most coming through the nose. The discharge was at first quite black, but free from odor. After washing with warm water, I threw in peroxid of hydrogen and then a four per cent. solution of boracic acid. After using the peroxid, the patient would complain of a stinging sensation for some time, and, I think, had I not had such a good opening it would have been better not to have used so much of it. Before dismissing the patient I was each time careful to cork the opening, to keep the secretions of the mouth out. I followed this treatment every day for about a week, without seeing any change. I then increased it to three times a day, and followed this up for about two weeks; at the end of that time I could scarcely detect any discoloration in the discharge. I then treated it once a day for a week, and then twice a week. It was but a short time before it commenced to heal—all pain subsided, swelling disappeared, and the patient got well. Could I have caused this disturbance extracting the tooth?—*Dental Register*.

